# Characterization of TgPuf1, a member of the Puf family RNA-binding proteins from *Toxoplasma gondii*

**DOI:** 10.1186/1756-3305-7-141

**Published:** 2014-03-31

**Authors:** Min Liu, Jun Miao, Tingkai Liu, William J Sullivan, Liwang Cui, Xiaoguang Chen

**Affiliations:** 1 Department of Pathogen Biology, School of Public Health and Tropical Medicine, Southern Medical University, Guangzhou, Guangdong 510515, China; 2 Department of Entomology, Pennsylvania State University, 501 ASI Building, University Park, PA 16802, USA; 3 Department of Pharmacology & Toxicology, Indiana University School of Medicine, Indianapolis, IN, USA; 4 Microbiology & Immunology, Indiana University School of Medicine, Indianapolis, IN, USA

**Keywords:** RNA binding protein, Puf, Subcellular localization, *Toxoplasma*

## Abstract

**Background:**

Puf proteins act as translational regulators and affect many cellular processes in a wide range of eukaryotic organisms. Although Puf proteins have been well characterized in many model systems, little is known about the structural and functional characteristics of Puf proteins in the parasite *Toxoplasma gondii*.

**Methods:**

Using a combination of conventional molecular approaches, we generated endogenous *TgPuf1* tagged with hemagglutinin (HA) epitope and investigated the TgPuf1 expression levels and localization in the tachyzoites and bradyzoites. We used RNA Electrophoretic Mobility Shfit Assay (EMSA) to determine whether the recombination TgPuf1 has conserverd RNA binding activity and specificity.

**Results:**

TgPuf1 was expressed at a significantly higher level in bradyzoites than in tachyzoites. TgPuf1 protein was predominantly localized within the cytoplasm and showed a much more granular cytoplasmic staining pattern in bradyzoites. The recombinant Puf domain of TgPuf1 showed strong binding affinity to two RNA fragments containing Puf-binding motifs from other organisms as artificial target sequences. However, two point mutations in the core Puf-binding motif resulted in a significant reduction in binding affinity, indicating that TgPuf1 also binds to conserved Puf-binding motif.

**Conclusions:**

TgPuf1 appears to exhibit different expression levels in the tachyzoites and bradyzoites, suggesting that TgPuf1 may function in regulating the proliferation or/and differentiation that are important in providing parasites with the ability to respond rapidly to changes in environmental conditions. This study provides a starting point for elucidating the function of TgPuf1 during parasite development.

## Background

The phylum *Apicomplexa* consists of single-celled eukaryotic parasites that are responsible for a variety of diseases in humans, pets and farm animals, and are thus of considerable medical and economic importance. Apicomplexan parasites are characterized by complex life cycles usually alternating between sexual and asexual stages involving different hosts. Among these parasites, the best known are *Plasmodium falciparum*, the causative agent of human malignant malaria and *Toxoplasma gondii*, responsible for toxoplasmosis in animals and humans. Both of these pathogens have evolved an obligate intracellular lifestyle, with growth, differentiation and replication taking place exclusively inside a protective parasitophorous vacuole within host cells. Unlike *Plasmodium*, *T. gondii* can infect a wide range of nucleated cells and differentiate into bradyzoites within tissue cysts that remain latent. Chronic infection with latent bradyzoite cysts is asymptomatic in immunocompetent individuals; however, upon host immunosuppression the parasite reconverts into its proliferative tachyzoite form, which causes severe tissue damage that can result in organ failure and death [1]. Understanding the molecular mechanisms underpinning the conversion of these life stages may identify novel molecular targets for treatment.

Translational control plays a critical role in the regulation of gene expression in most organisms. Compared with transcriptional regulation, translational control of gene expression allows the cell to respond more rapidly to external stimuli [2]. The Puf family RNA-binding proteins (RBPs) modulate mRNA expression in a wide variety of eukaryotic species [3]. PUF proteins execute translation control by binding to specific ribonucleotide sequences called Puf-binding element (PBE), which typically reside in the 3’ untranslated region (3′ UTR) of target mRNAs. The signature feature of the Puf proteins is a highly conserved core RNA-binding domain, referred to as the Puf domain, which almost always contains eight copies of a similar α-helical repeat flanked by one imperfect pseudo-repeat at each end. The Puf domains of Puf proteins from different species are incredibly well conserved, whereas sequences outside the Puf domain vary significantly [4]. The number of Puf genes in each organism is also variable. For example, the *Drosophila*, human, yeast, and *C. elegans* genomes encode one, two, six and eleven Puf genes, respectively [3]. While the canonical role of PUFs is translational repression [3,5], recent evidence suggests that they can contribute to the activation of mRNA expression in some species [6-9]. Furthermore, some have reported that PUFs contribute to the targeting of mRNAs to specific subcellular locations to provide spatial control of expression [10-15]. To date, the functions of Puf proteins have been elucidated during the developmental processes of a number of organisms. Puf proteins have diverse functions, but they appear to share a common, probably ancestral, role in each species that involves promoting proliferation of cells and repressing differentiation [3]. In the protozoan parasite *Trypanosoma brucei*, Puf1 is essential for cell viability [16]. In *Plasmodium*, two conserved Puf proteins are preferentially expressed in gametocyte and sporozoite stages [17,18]. Notably, Puf2 protein appears to play important roles in the stage transition of the malaria parasites. Genetic knockout of the *Puf2* gene in *P. falciparum* and *P. berghei* promotes differentiation of gametocytes and elevates the male/female sex ratio [19,20]. In *P. berghei* sporozoites, Puf2 knockout (KO) parasites experience premature transformation of the sporozoites into forms resembling early intra-hepatic stages while the sporozoites are still inside the salivary glands of the mosquito [19,21]. Recently, it has been revealed that PfPuf2 regulates the translation of a number of transcripts in gametocytes, including two genes encoding the transmission-blocking vaccine candidates Pfs25 and Pfs28 [20]. Altogether, these studies have shed light on the molecular mechanisms by which Puf family proteins regulate mRNA translation.

Translational control contributes to gene regulation in *Apicomplexa*, particularly in the context of stage differentiation [22]. For instance, the transcript level of *bsr4* transcript is equally abundant in both tachyzoites and bradyzoites, but the bsr4 protein is up-regulated only in bradyzoites [23]. Additionally, the phosphorylation of eukaryotic initiation factor-2α, which induces translational control, has been linked to microbial latency in *T. gondii*[24]. The interesting functions of Puf proteins in regulating stage transition in *Plasmodium* parasites have prompted us to investigate the Puf homologs in *T. gondii*. Here, we performed molecular characterization of TgPuf1 in *T. gondi* and determined its expression, cellular localization and *in vitro* RNA-binding activity of the recombinant protein*.* Our results indicate that gene regulation via translational control has an additional level of complexity that involves the 3′UTR in this important group of parasites.

## Methods

### Parasite culture

The virulent RH∆Ku80 and avirulent Pru∆Ku80 (Prugniaud) strains of *T. gondi* were maintained by serial passage in human foreskin fibroblasts (HFF) cultivated in Dulbecco’s modified Eagle medium (DMEM) supplemented with 1% (v/v) heat-inactivated fetal bovine serum (FBS) and 25 μg/L gentamicin antibiotic (Life Technologies). To induce bradyzoite formation, ~50,000 tachyzoites were inoculated onto confluent HFF monolayers in T25 flasks with culture medium. Two to three hours post infection, the culture medium was replaced with a pH 8.2 medium, which was replaced daily.

### Phylogenetic comparisons

A total of 47 GenBank entries with complete Puf domains were retrieved for phylogenetic analysis. The Puf domains of TgPufs were trimmed and used to generate the data matrix to infer the phylogenetic relationships among Puf family members. Multiple alignment was performed using the CLUSTALW program (http://www.ebi.ac.uk/clustalw) and the phylogenetic tree was constructed by the neighbor-joining (NJ) method with bootstrap analysis (1000 pseudo-replications) using the MEGA 4 program (http://www.megasoftware.net).

### Expression of recombinant TgPuf1 Puf domain in *Escherichia coli*

To express the conserved RNA-binding domain of TgPuf1 in bacteria, PCR was performed with *T. gondii* cDNA using two primers (CG*GGATCC*AGAAAAGGCGACTCAAAAG and ATAAGAAT*GCGGCCGC*GTCACTGAAACCTGAGATG) designed to clone at the *Bam*HI and *Not*I sites of the expression vector pGEX-6P-1 (GE Healthcare). The TgPuf1 Puf domain was expressed in *E.coli* strain BL21 (DE3) as a fusion to the carboxyl-terminus of glutathione S-transferase (GST). Bacteria were grown overnight at 37°C, diluted 1:100 in fresh media and grown to an OD_600_ value of 0.6. Induction was performed by the addition of 0.1 mM of IPTG and incubated for 4 h. Recombinant protein was purified from 1 L culture using glutathione Sepharose-4B (GE Healthcare) and eluted with 50 mM Tris–HCl (pH 8.0) and 10 mM reduced glutathione. Purified recombinant TgPuf1 (rTgPuf1) protein was dialyzed extensively in phosphate-buffered saline (PBS, pH 7.0) and used for immunization in rabbits for antibodies and for *in vitro* RNA binding assay.

### Plasmid construction and parasite transfection

A *Toxoplasma* clone stably expressing TgPuf1 tagged at its C-terminus with the 3X hemagglutinin (HA) epitope was generated by targeting the endogenous *TgPuf1* locus using homologous recombination. RH∆Ku80 genomic DNA was used to amplify a 1.3-kb fragment of the Puf1 3′ end using primers Puf1HA_F (5′-*TACTTCCAATCCAATTTAATGC*GTATGCGAACTATGGTAAGACT-3′) and Puf1HA_R (5′-*TCCTCCACTTCCAATTTTAGC*CATCCCATCGACAGCAATC-3′) that contained ligation-independent cloning sequences (italics). This Puf1 fragment was inserted into the pLIC_HAx3_DHFRTs endogenous tagging vector such that the TgPuf1 coding sequence was fused in frame with the epitope coding region. The pLIC_Puf1HAx3_DHFRTs construct was confirmed by sequencing. For transfection, 30 μg of the pLIC_Puf1HAx3_DHFRTs plasmid was linearized by overnight digestion with *Blp*I within the Puf1 homologous region and ethanol precipitated. RH∆Ku80 and Pru∆Ku80 tachyzoites were transformed with the linearized construct by electroporation, and after overnight growth in HFF, parasite cultures were selected with 1.0 μM pyrimethamine [25]. Drug-resistant parasites were cloned by limiting dilution and screened by Western blot and immunofluorescence for expression of HA-tagged TgPuf1.

### Western blot

To study TgPuf1 protein expression, equal amounts of the parasite lysates (25 μg) of tachyzoites and bradyzoites were separated by SDS/PAGE (8%) and transferred to nitrocellulose membranes. Bradyzoites were induced by alkaline-stress for 12 days. To isolate bradyzoites, infected cells were scraped from the flask and passed through an 18G needle for 10 times, and bradyzoites were purified from host cell debris by filtration through a 25 mm Nuclepore Track-Etched Polycarbonate Membrane circle with a 3.0 μm pore size (GE Healthcare) into a conical tube. The parasites were pelleted by centrifugation and washed with cold PBS at 4°C. Western blot was carried out using rat anti-HA antibodies (Roche) (1:2,000) or rabbit anti-rTgPuf1 antiserum (1,1000) as the primary antibodies and horseradish peroxidase-conjugated goat anti-rabbit or anti-rat IgG (1:3,000) as the secondary antibodies. Antibodies to *Toxoplasma* BAG1 (1:1,000) were used to detect protein expression in bradyzoites. *Toxoplasma* β-tubulin expression detected by specific polyclonal antibodies (1:1,000) served as a protein loading control. The results were visualized with the ECL detection system (GE Healthcare). The density of bands detected in Western blot was analyzed by ImageJ software and normalized with the β-tubulin loading control as the ratio of TgPuf1/β-tubulin. This experiment was repeated three times, and the expression levels of TgPuf1 between bradyzoites and tachyzoites were compared by T-test.

### Indirect Immunofluorescent assay (IFA)

For IFA, infected HFF monolayers grown on coverslips were fixed in 4% paraformaldehyde for 20 min at room temperature. They were then permeablized for 10 min in PBS containing 3% BSA and 0.2% Triton X-100 and blocked for 1 h in PBS with 3% BSA. They were first probed with rabbit anti-HA antibody (Sigma) (1:500) and anti-BAG1 antibodies (1:100). Secondary antibodies were FITC-labeled anti-rabbit IgG (Sigma) and TRITC-labeled anti-mouse IgG (Sigma). Fluorescent images were obtained with a Nikon ECLIPSE E600 epifluorescence microscope.

### In vitro RNA binding assay

Electrophoretic mobility shift assay (EMSA) was performed using the Light Shift Chemiluminescent RNA EMSA kit (Pierce). Briefly, each 20 μl of reaction contained 2 μg tRNA for blocking non-specific RNA-protein interactions, EMSA binding buffer, 20 units of RNase inhibitor, 5% glycerol, rTgPuf1, and biotinylated RNA oligos with or without cold competitors. The artificial Puf target RNAs included the *Drosophila hunchback* (*hb*) Nanos Response Element (NRE) sequence AUUAUUUU*GUUGU*CGAAA*AUUGU*ACAUAAGCC [17] and the *pfs28* 3′ UTR sequence (Pfs28 RNA1) GAAAUGUUCUUU*UGUAAUUA*UAUUUUGUUCGAUGAUUC [20], where the PBEs essential for Puf binding are in italics. A Pfs28 RNA1Moligo, in which the UGU sequence in the PBE of Pfs28 RNA1 was mutated to UCC, was used to determine whether this would interfere with TgPuf1 binding [20]. These oligos were synthesized as biotin-labeled RNA fragments (Integrated DNA Technologies). In a 20 μl reaction, 2.5 nM of an RNA oligo and different concentrations of rTgPuf1 (0.78 – 400 nM) were incubated at room temperature for 20 min. Cold competitor (unlabeled) RNAs were included at 5 X, 50 X and 100 X concentrations of the biotinylated RNAs to demonstrate binding specificity. The reactions were electrophoresed on a 5% native acrylamide/8 M urea gel and transferred to a nylon membrane. The bands of labeled oligos were detected using the Chemilumescent Nucleic Acid Detection Module (Pierce). Each experiment was repeated three times and the average *K*_d_ values were estimated by fitting the curves to the mean percentages of the total bound RNA, which were determined by densitometry using the Quantity One 1-D Analysis Software (BioRad).

## Results

### *Toxoplasma* encodes two putative Puf proteins

**Figure 1:**
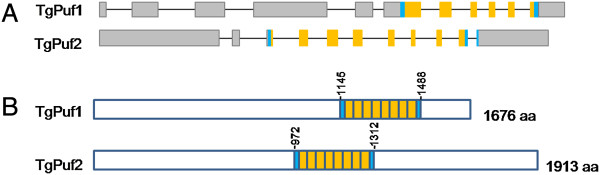
**TgPuf Pumilio homology domain (PUM-HD) organization. (A)** A schematic representation of the genomic structure of *TgPuf* loci (not to scale). Exons are indicated as boxes and introns as solid lines. The conserved RNA-binding domain (RBD) is shown as yellow boxes and the two flanking imperfect pseudo-repeats are shown as blue boxes. **(B)** The domain organization of predicted TgPuf1 and TgPuf2 proteins. RBDs for each protein are shown with eight repeats (yellow boxes) and two flanking imperfect pseudo-repeats (blue boxes) (not to scale). Puf domain of TgPuf1 is located near the C-terminus (1145–1488 aa), whereas the Puf domain of TgPuf2 is located close to the center of the predicted protein (972–1312 aa).

A BLASTP search of the *T.gondii* genome in the ToxoDB with the conserved Puf domain of *Plasmodium* identified two Puf homologs (TGME49_260600 and TGME49_318350). A phylogenetic tree was constructed based on CLUSTALW alignment of 47 GenBank Puf sequences with complete Puf domains and the two TgPuf sequences (Additional file 1: Figure S1). Based on their degrees of homology to the *Plasmodium* Pufs, the TGME49_260600 and TGME49_318350 genes are designated as TgPuf1 and TgPuf2, respectively. TgPuf1 is located in chromosome VIIb and is 13,034 bp in length, containing ten introns (Figure 1A). TgPuf1 encodes a predicted protein of 1676 amino acids (aa) with the Puf domain located near the carboxyl terminus (1145–1488 aa) (Figure 1B). TgPuf2 is located in chromosome IV and is 9,773 bp long, also containing ten introns (Figure 1A). TgPuf2 encodes a predicted protein of 1913 aa and the Puf domain is at the center of the protein (972–1312 aa) (Figure 1B). The two putative *T. gondii* Puf proteins share limited homology (~ 26% identity), and the homology is restricted to the Puf domains.

**Figure 2:**
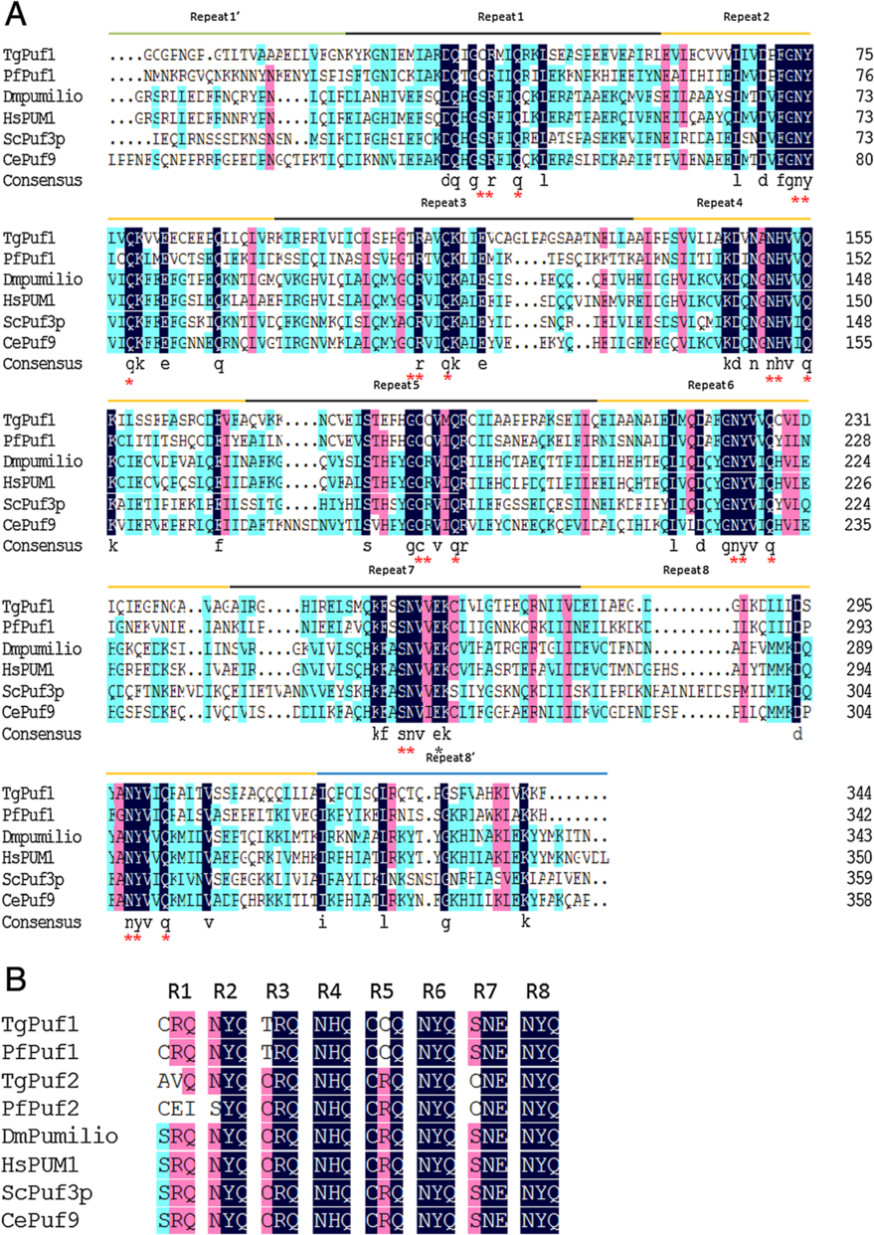
**Sequence alignment of Puf domains (8 imperfect repeats) in selected Puf proteins. (A)** Sequences were from *T.gondii* (TgPuf1), *P.falciparum* (PfPuf1), *D.melanogaster* (DmPumilio, CAA44474.1), *Homo sapiens* (HsPUM1, NP 001018494.1), *S.cerevisiae* (ScPuf3p, NP 013088.1) and *C.elegans* (CePuf9, NP 508980.2). Identical amino acids are highlighted in black and similar residues are shown in pink and blue (less similar). * indicates amino acids that are putative RNA contact sites. **(B)** Sequence alignment of amino acid triplets at the positions 12, 13 and 16 in each Puf repeat (R1 to R8).

Like other Puf members, the Puf domain of TgPuf1 is composed of eight tandem imperfect repeats of ~36 aa plus two flanking imperfect pseudo repeats (Figure 2A). These flanking regions resemble half-repeats and are therefore called repeat 1′ and repeat 8′, respectively [26]. The TgPuf1 Puf domain has the highest sequence homology to the PfPuf1 Puf domain with 44% amino acid identity. Structure analysis of Puf proteins from several model species determined that the residues at positions 12 and 16 of each Puf repeat bind the Watson-Crick edge of each RNA base via hydrogen or van der Waals contacts, while the position 13 residue makes a stacking interaction [27]. Alignment of these aa triplets in the Puf repeats from *Toxoplasma* and several model organisms revealed a high degree of conservation (Figure 2B). Specifically, the aa triplets of TgPuf1 and PfPuf1 repeats are completely conserved, and differed from those in the model organisms at three positions. Repeat 1 in TgPuf1 possesses a cysteine at position 12 (forming a CRQ triplet); this CRQ triplet is also found in repeat 1 in some fungal, protozoan, and plant Puf proteins [28,29]. Repeat 3 of TgPuf1 possesses a threonine at position 12 (TRQ) and repeat 5 possesses a cysteine at position 13 (CCQ). These TRQ and CCQ triplets are conserved with some plant Puf proteins but are different from SRQ and CRQ in the human PUM1 [29]. In comparison, some unconventional triplets are present in the TgPuf2 and PfPuf2 repeats 1.

### Expression of the recombinant TgPuf1 Puf domain

**Figure 3:**
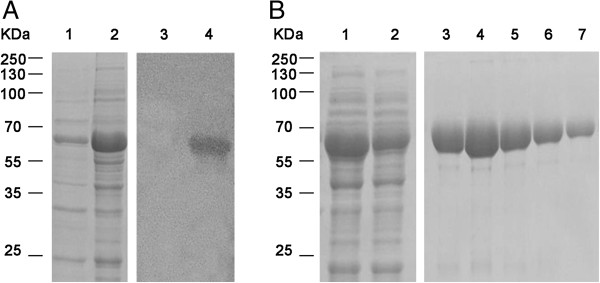
**Expression and purification of the rTgPuf1 Puf domain in *E. coli*. (A)** Left panel shows a Coomassie Blue stained gel. Lane 1 and 2 shows the pellet and supernatant of lysates of induced pGEX-6P-1-TgPuf1-PUM-HD/BL21. Right panel is the immunoblot with anti-GST antibodies, which detected rTgPuf1 expression in uninduced (lane 3) and IPTG induced BL21 cells (lane 4). **(B)** Purification of rTgPuf1. Lane 1, lysate of induced BL21 cells; lane 2, lysate passed through a GST column; lane 3–7, elution with 50 mM Tris–HCl and 10 mM glutathione (pH 8.0).

To investigate whether TgPuf1 has RNA binding activity, the putative Puf domain of TgPuf1 (354 aa) plus short sequences on each side corresponding to the Pum RNA-binding domain was expressed in a bacterial expression system. The GST-tagged rTgPuf1 was affinity-purified and confirmed by immunoblotting with the anti-GST antibody (Figure 3A). The protein size (68 kDa) was consistent with the predicted molecular size of the rTgPuf1 Puf domain (42 kDa) plus the GST tag (26 kDa).

### TgPuf1 is expressed in both tachyzoite and bradyzoite

**Figure 4:**
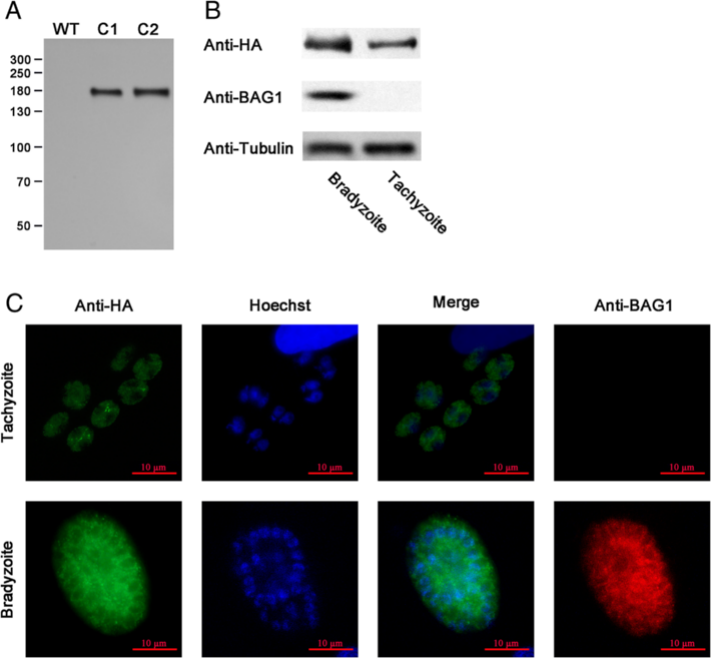
**TgPuf1 expression and subcellular localization in the Pru∆Ku80 parasite. (A)** Confirmation of the C-terminal HA x 3-tagging of the endogenous TgPuf1 locus. Two clones (C1 and C2) were probed with the anti-HA antibody. Lysate from wild-type Pru strain parasite was included as a HA-negative control. **(B)** Expression of TgPuf1 in tachyzoites and bradyzoites. The two stages of the parasite were differentiated by antibodies against BAG1, a protein expressed specifically in the bradyzoite stage. Anti-β-tubulin antibody served as a protein loading control. **(C)** Subcellular localization of TgPuf1 in tachyzoites and bradyzoites.

To study stage-specific expression and subcellular localization of the TgPuf1 protein in tachyzoites and bradyzoites, we transfected the Pru∆Ku80 parasite strain and tagged the C-terminus of the endogenous TgPuf1 with a 3XHA tag. Successful tagging of the endogenous TgPuf1 protein was confirmed by IFA and Western blot. Two clones with the HA tag integrated at the *TgPuf1* locus were selected for protein expression analysis. Western blot using the anti-HA antibodies detected a specific protein band of 175 kDa, consistent with the predicted size of the TgPuf1-HA fusion protein (Figure 4A), whereas this protein was not detected in the control Pru∆Ku80 parasites. Quantification of the protein bands detected in Western blots showed that the ratio of TgPuf1/β-tubulin protein levels was significantly increased in cultures enriched with bradyzoites (1.04 ± 0.09) compared to tachyzoites (0.55 ± 0.05)(*P* = 0.001, T test) (Figure 4B). Tagging of TgPuf1 in the RH∆Ku80 strain revealed a similar expression pattern of TgPuf1 (Additional file 2: Figure S2A, B). Furthermore, probing wild-type parasite strains with anti-tgPuf1 antibodies also indicated increased expression of TgPuf1 in bradyzoites (Additional file 3: Figure S3).

### Subcellular localization of TgPuf1

IFA with anti-HA antibodies detected TgPuf1 protein in the cytoplasm, consistent with its function in translation control. In bradyzoites induced by alkaline-stress, the TgPuf1 protein showed a much more granular cytoplasmic staining pattern. Such punctate cytoplasmic structures were more obvious in bradyzoites (Figure 4C, Additional file 2: Figure S2), whereas they had a relatively uniform distributionin the cytoplasm of tachyzoites (Figure 4C, Additional file 2: Figure S2).

### In vitro binding activity of the rTgPuf1

**Figure 5:**
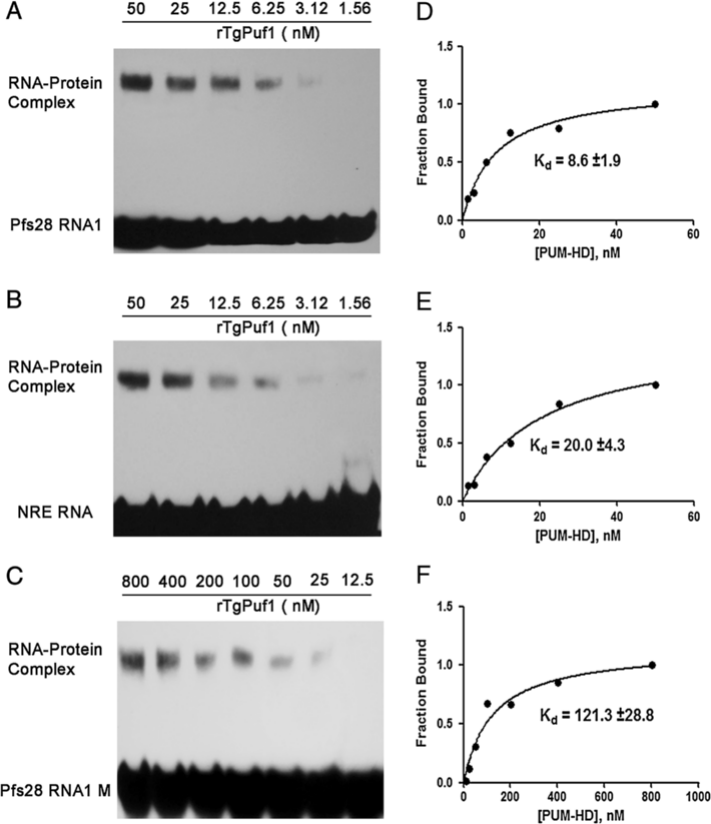
**RNA binding analysis of rTgPuf1 PUM-HD.** EMSA titration of rTgPuf1 PUM-HD binding toPfs28 RNA1 **(A)**, *hb* NRE RNA **(B)**, and Pfs28 RNA1M **(C)**. In Pfs28 RNA1M, the UGU sequence in the putative PBE of Pfs28 RNA1 was mutated to UCC. **(D)**, **(E)** and **(F)** Quantitation of dissociation constant (Kd) values based on EMSA analysis from **(A)**, **(B)** and **(C)**, respectively.

**Figure 6:**
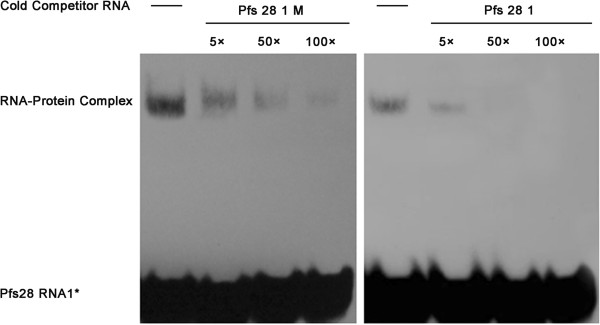
**TgPuf1 binds conserved RNA motifs.** Competition EMSA shows the specificities of rTgPuf1 binding to Pfs28 RNA1 but not to mutant Pfs28 RNA1. Competitor RNAs were added in reactions at 5X, 50X and 100X of the biotinylated probe (labeled with an asterisk).

Both the human and mouse recombinant Puf proteins produced in bacteria bind to the *Drosophila* NRE sequence *in vitro*[30,31], which suggests that *hb* NREs may be used as artificial targets to study the binding activity of other Puf family proteins, especially when their authentic target mRNAs are unknown. Homology analysis results showed that TgPuf domains are more related to the Puf domains in PfPufs. To determine whether the rTgPuf1 had conserved RNA binding activity, EMSA was performed using NRE, Pfs28 RNA1 and Pfs28 RNA1M as potential target RNAs. EMSA experiments demonstrated that rTgPuf1 bound more efficiently to NRE and Pfs28 RNA1, and significantly less efficiently to Pfs28 RNA1M (Figure 5A,B,C). Titration of the binding efficacy showed that rTgPuf1 bound to the Pfs28 RNA1 and *hb* NRE with an apparent dissociation constant of 8.6 ± 1.9 nM and 20.0 ± 4.3 nM, respectively (Figure 5D, E). In contrast, the binding affinity of the protein to the Pfs28 RNA1M with the mutant PBE was significantly reduced with a *K*_d_ value of 121.3 ± 28.8 nM (~14-fold reduction in affinity) (Figure 5F). To further corroborate that rTgPuf binding to the Pfs28 RNA1 was specific, competition experiments with unlabeled RNA competitors of Pfs28 RNA1 and Pfs28 RNA1M was performed. Binding to labeled Pfs28 RNA1 was efficiently competed by its cognate cold RNA but not by the mutant Pfs28 RNA1M (Figure 6), indicating that rTgPuf1 binding to the PBE present in the Pfs28 RNA1 was specific.

## Discussion

Translational regulation of gene expression plays an important role in the development of diverse eukaryotes. In many cases, post-transcriptional regulation requires *cis*-acting sequences located in either the 3′ or 5′ UTRs of the transcript. We have shown here that *T.gondii* possesses two distinct Puf members, which share limited sequence similarity, suggesting they might regulate different RNA repertoires and have different functions. Interestingly, TgPuf1 and TgPuf2 are more homologous to their respective Puf1 and Puf2 genes in *Plasmodium*, suggesting that the duplication of Puf genes in these two Apicomplexan parasites occurred earlier before the divergence of these parasite taxa. In the malaria parasite *P. berghei*, only PbPuf2 are found to regulate the stage-transition in sporozoites, whereas deletion of *PbPuf1* had no effects on this process [1,2,19,28]. In *P. falciparum* both Puf proteins are abundantly expressed in gametocytes and Puf2 plays an important role during gametocytogenesis [32]. In *Toxoplasma*, TgPuf1 appeared to be more abundantly expressed in bradyzoites at the protein level, suggesting that TgPuf1 protein may also function during the tachyzoite-bradyzoite transformation. Future study will be directed to decipher the potential role of TgPuf1 in regulating stage transition through gene disruption analysis.

The PUF domain contains eight PUM repeats, each containing three α-helices packed together in a curved structure. RNA is bound as an extended strand to the concave surface of the PUF domain with the bases contacted by protein side chains [33]. Specifically, the eight bases of the target RNA, 1–8, are contacted by protein repeats 8–1, with the critical UGU sequence recognized by repeats 8, 7 and 6, respectively [33]. Here we showed that the rTgPuf1 PUM domain has the conserved RNA binding activity to canonical target RNAs and the binding depends on the presence of the essential UGU motif. In line with other reports, mutations in the UGUR sequence abolishes or significantly interferes with the binding [30,31,34-38]. A search of the *Toxoplasma* genome for the presence of the PBE sequence identified 130,571 UGUX_3_UA motifs, which remain to be determined as Puf binding targets.

In accordance with the primary role of Puf proteins, Puf proteins are predominantly localized within the cytoplasm of cells. Two exceptions are *T. brucei* Puf7, which is localized in the nucleolus [39], and *S. cerevisiae* Puf6p, which is present in both the cytoplasm and nucleus [40]. TgPuf1 is localized in the cytoplasm and it forms punctate cytoplasmic structures in bradyzoites. These structures are reminiscent of “stress granules” or “processing bodies (p-bodies)” formed upon exposure of the cells to stress conditions [41,42]. Stress granules are large cytoplasmic aggregates, where mRNAs stalled at translation initiation are stored. They contain numerous RBPs, mRNA, the 40S ribosomal subunit and a number of initiation factors [41,42]. Whereas stress granules are rarely found in growing cells, they are induced rapidly after exposure to many types of stress. P-bodies are typically found in growing cells, however, they become larger and more numerous upon exposure to stress, and can be observed to physically interact with stress granules [41,42]. Stress granules and P-bodies have been found to contain a large number of RBPs, including Puf proteins [41-43]. Given the localization of human PUM1 and PUM1 to cytoplasmic stress granules, the punctate staining patterns of TgPuf1 suggest that they might be localized to similar granules, although it still requires co-localization confirmation with a marker for the stress granules or P-bodies [10,44]. Interestingly, several target mRNAs protected from translation and degradation in the *P. berghei* gametocytes are bound to the DOZI RNA helicase complex, which is apparently devoid of Puf proteins [45,46]. Future work is necessary to elucidate the biological roles, spatial and temporal regulation, interaction partners, and regulated biological pathways of TgPuf proteins.

## Conclusions

We have shown here that TgPuf1 has conserved RNA-binding activity and specificity towards the Puf-binding elements. It appears to be expressed differentially in tachyzoites and bradyzoites, suggesting that TgPuf1 may function in regulating the proliferation or/and differentiation, which might be important in providing parasites with the ability to respond rapidly to changes of environmental conditions.

## Competing interests

The authors declare that they have no competing interests.

## Authors’ contributions

ML carried out the laboratory work, analysed the data and prepared initial draft of the manuscript and its figures. JM and TL contributed in the experimental technical guidance. JM participated in some figures editing. WJSJ provided some study materials and commented on the final manuscript. LC and XC conceived and coordinated the study and revised the manuscript. All authors read and approved the final version of the manuscript.

## Supplementary Material

Additional file 1: Figure S1 A phylogenetic tree showing the relationship between the amino acid sequences of Puf members. The tree includes all members from *T. gondii* (Tg) and *P.falciparum* (Pf), and representative members from mouse, human (HsPum), *Xenopus*, *Drosophila* (DrPumilio), *Caenorhabditis elegans* (Ce), *Saccharomyces cerevisiae* (Sc), *Leishmania*, *Trypanosoma*, *Arabidopsis,* and *Neurospora*. Only the PUM-HDs were used for alignment. TgPufs are highlighted with arrows. Click here for file

Additional file 2: Figure S2 TgPuf1 expression and subcellular localization in the RH∆Ku80 parasite. (A) Confirmation of the C-terminal HA x 3-tagging of the endogenous TgPuf1 locus. Two clones (C5 and C9) were probed with the anti-HA antibody. Lysate from wild-type RH strain parasite was included as a HA-negative control. The lower, cross-reacting bands were probably degradation products of the tagged TgPuf1 protein, and they were detected in transfected Pru∆Ku80 lines after longer exposure of the film. (B) Expression of TgPuf1 in tachyzoites and bradyzoites. The two stages of the parasite were differentiated by antibodies against BAG1, a protein expressed specifically in the bradyzoite stage. Anti-β-tubulin antibody served as a protein loading control. (C) Subcellular localization of TgPuf1 in tachyzoite and bradyzoite. Click here for file

Additional file 3: Figure S3 Lysate of *T. gondii* parasites immunoblotted with anti-rTgPuf1 polyclonal antibodies. Left panel: Expression of TgPuf1 in untransfected RH∆Ku80 tachyzoites. Parasite lysates were probed with preimmune and immune serum against rTgPuf1. The arrow indicates the predicted TgPuf1 protein, while the other bands are probably cross-reacting proteins. Right panel: Expression of TgPuf1 in tachyzoites and bradyzoites was determined by immunoblotting with anti-rTgPuf1 polyclonal antibody. The two stages were differentiated by antibodies against bradyzoite-specific BAG1. Anti-β-tubulin antibody served as a protein loading control. Click here for file
