# Supplementary material for: Characterization of TgPuf1, a member of the Puf family RNA-binding proteins from Toxoplasma gondii
Source: Parasit Vectors. 2014 Mar 31;7:141. doi: 10.1186/1756-3305-7-141 (PMC3997814; doi:10.1186/1756-3305-7-141)
Supplement: Additional file 1: Figure S1 — A phylogenetic tree showing the relationship between the amino acid sequences of Puf members. The tree includes all members from T. gondii (Tg) and P.falciparum (Pf), and representative members from mouse, human (HsPum), Xenopus, Drosophila (DrPumilio), Caenorhabditis elegans (Ce), Saccharomyces cerevisiae (Sc), Leishmania, Trypanosoma, Arabidopsis, and Neurospora. Only the PUM-HDs were used for alignment. TgPufs are highlighted with arrows. [file 1756-3305-7-141-S1.pptx]

## Slide 1
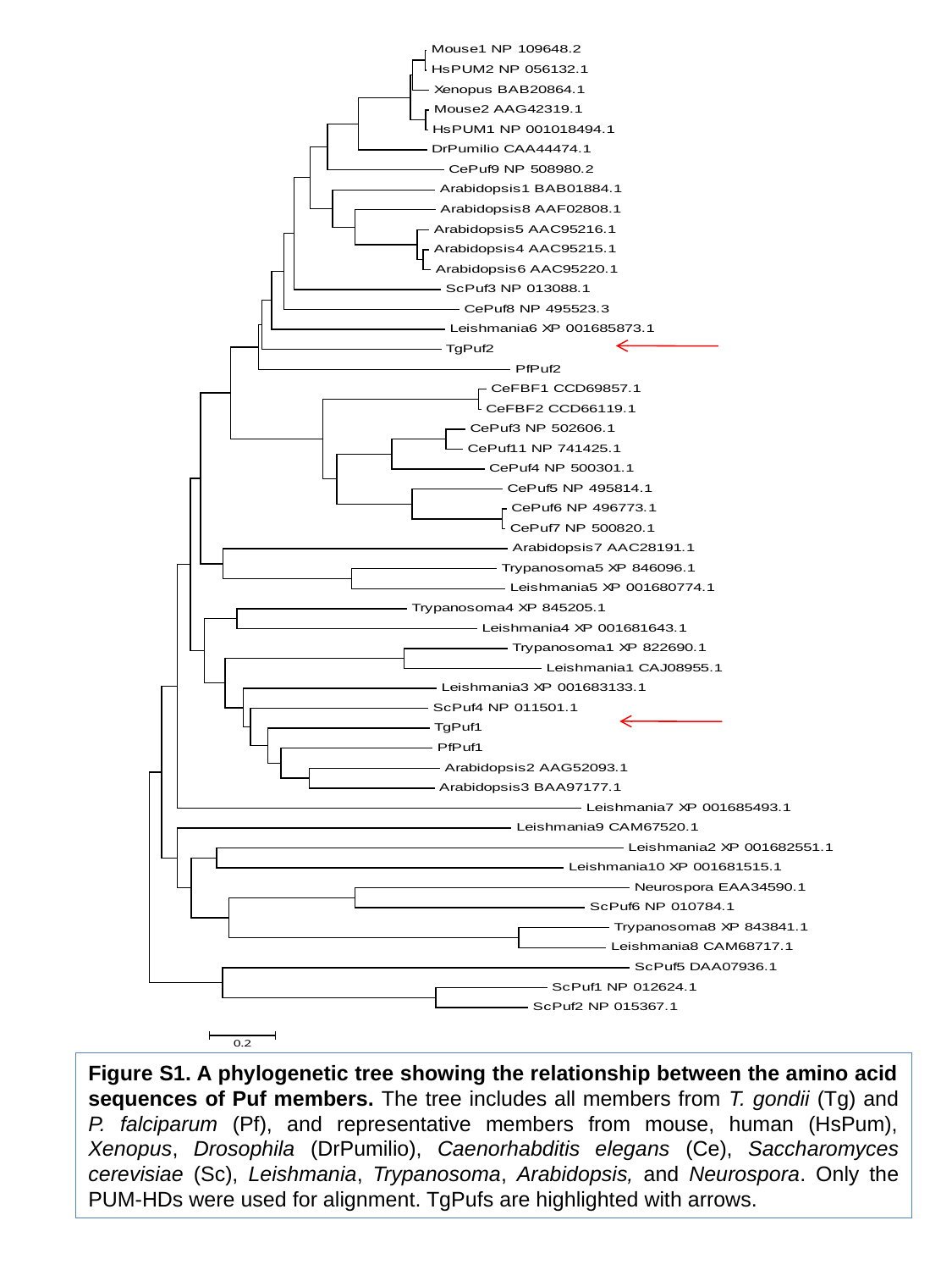

Figure S1. A phylogenetic tree showing the relationship between the amino acid sequences of Puf members. The tree includes all members from T. gondii (Tg) and P. falciparum (Pf), and representative members from mouse, human (HsPum), Xenopus, Drosophila (DrPumilio), Caenorhabditis elegans (Ce), Saccharomyces cerevisiae (Sc), Leishmania, Trypanosoma, Arabidopsis, and Neurospora. Only the PUM-HDs were used for alignment. TgPufs are highlighted with arrows.
