# Supplementary material for: Characterization of TgPuf1, a member of the Puf family RNA-binding proteins from Toxoplasma gondii
Source: Parasit Vectors. 2014 Mar 31;7:141. doi: 10.1186/1756-3305-7-141 (PMC3997814; doi:10.1186/1756-3305-7-141)
Supplement: Additional file 2: Figure S2 — TgPuf1 expression and subcellular localization in the RH∆Ku80 parasite. (A) Confirmation of the C-terminal HA x 3-tagging of the endogenous TgPuf1 locus. Two clones (C5 and C9) were probed with the anti-HA antibody. Lysate from wild-type RH strain parasite was included as a HA-negative control. The lower, cross-reacting bands were probably degradation products of the tagged TgPuf1 protein, and they were detected in transfected Pru∆Ku80 lines after longer exposure of the film. (B) Expression of TgPuf1 in tachyzoites and bradyzoites. The two stages of the parasite were differentiated by antibodies against BAG1, a protein expressed specifically in the bradyzoite stage. Anti-β-tubulin antibody served as a protein loading control. (C) Subcellular localization of TgPuf1 in tachyzoite and bradyzoite. [file 1756-3305-7-141-S2.pptx]

## Slide 1
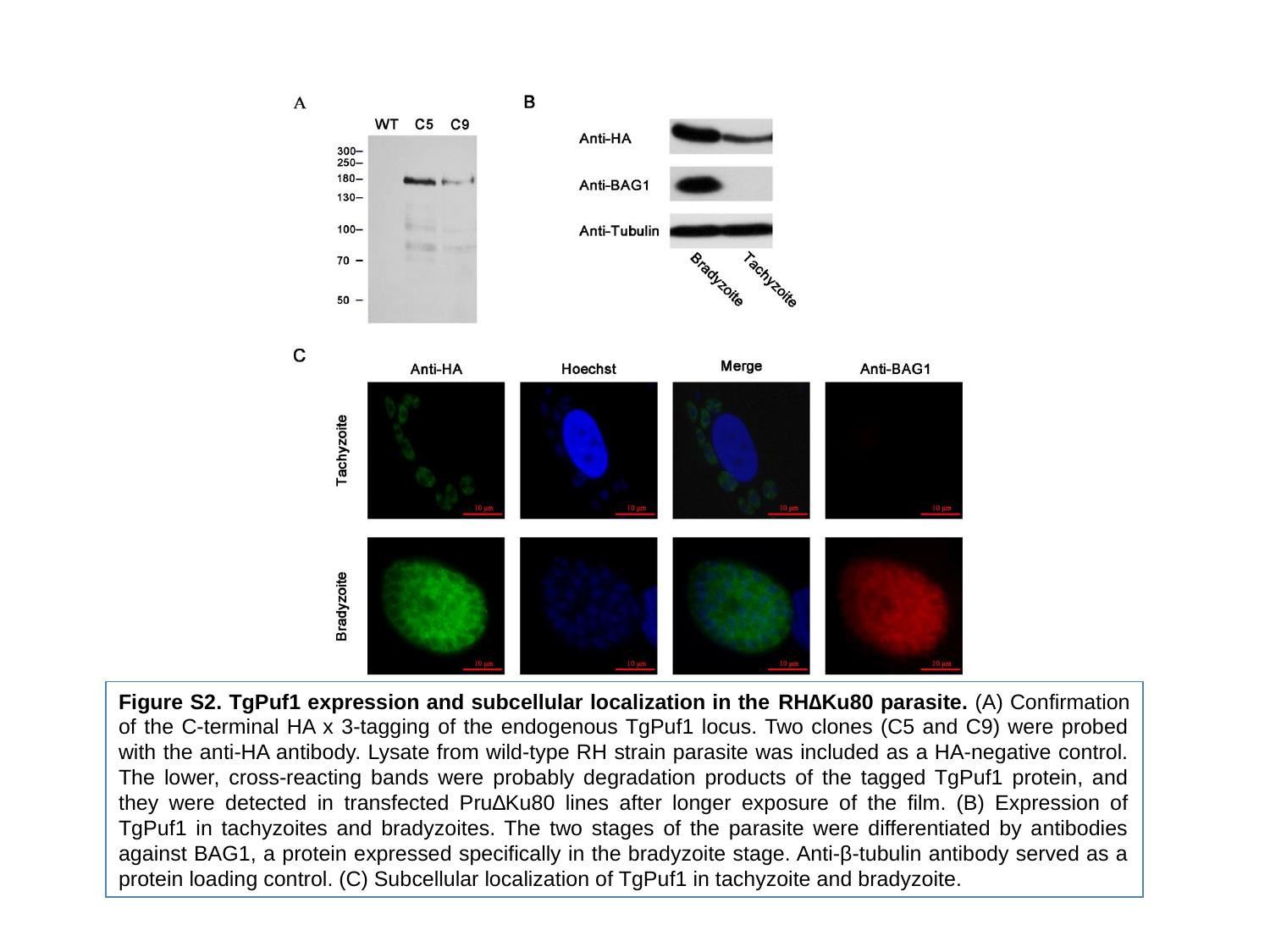

Figure S2. TgPuf1 expression and subcellular localization in the RH∆Ku80 parasite. (A) Confirmation of the C-terminal HA x 3-tagging of the endogenous TgPuf1 locus. Two clones (C5 and C9) were probed with the anti-HA antibody. Lysate from wild-type RH strain parasite was included as a HA-negative control. The lower, cross-reacting bands were probably degradation products of the tagged TgPuf1 protein, and they were detected in transfected Pru∆Ku80 lines after longer exposure of the film. (B) Expression of TgPuf1 in tachyzoites and bradyzoites. The two stages of the parasite were differentiated by antibodies against BAG1, a protein expressed specifically in the bradyzoite stage. Anti-β-tubulin antibody served as a protein loading control. (C) Subcellular localization of TgPuf1 in tachyzoite and bradyzoite.
