# Supplementary material for: Characterization of TgPuf1, a member of the Puf family RNA-binding proteins from Toxoplasma gondii
Source: Parasit Vectors. 2014 Mar 31;7:141. doi: 10.1186/1756-3305-7-141 (PMC3997814; doi:10.1186/1756-3305-7-141)
Supplement: Additional file 3: Figure S3 — Lysate of T. gondii parasites immunoblotted with anti-rTgPuf1 polyclonal antibodies. Left panel: Expression of TgPuf1 in untransfected RH∆Ku80 tachyzoites. Parasite lysates were probed with preimmune and immune serum against rTgPuf1. The arrow indicates the predicted TgPuf1 protein, while the other bands are probably cross-reacting proteins. Right panel: Expression of TgPuf1 in tachyzoites and bradyzoites was determined by immunoblotting with anti-rTgPuf1 polyclonal antibody. The two stages were differentiated by antibodies against bradyzoite-specific BAG1. Anti-β-tubulin antibody served as a protein loading control. [file 1756-3305-7-141-S3.pptx]

## Slide 1
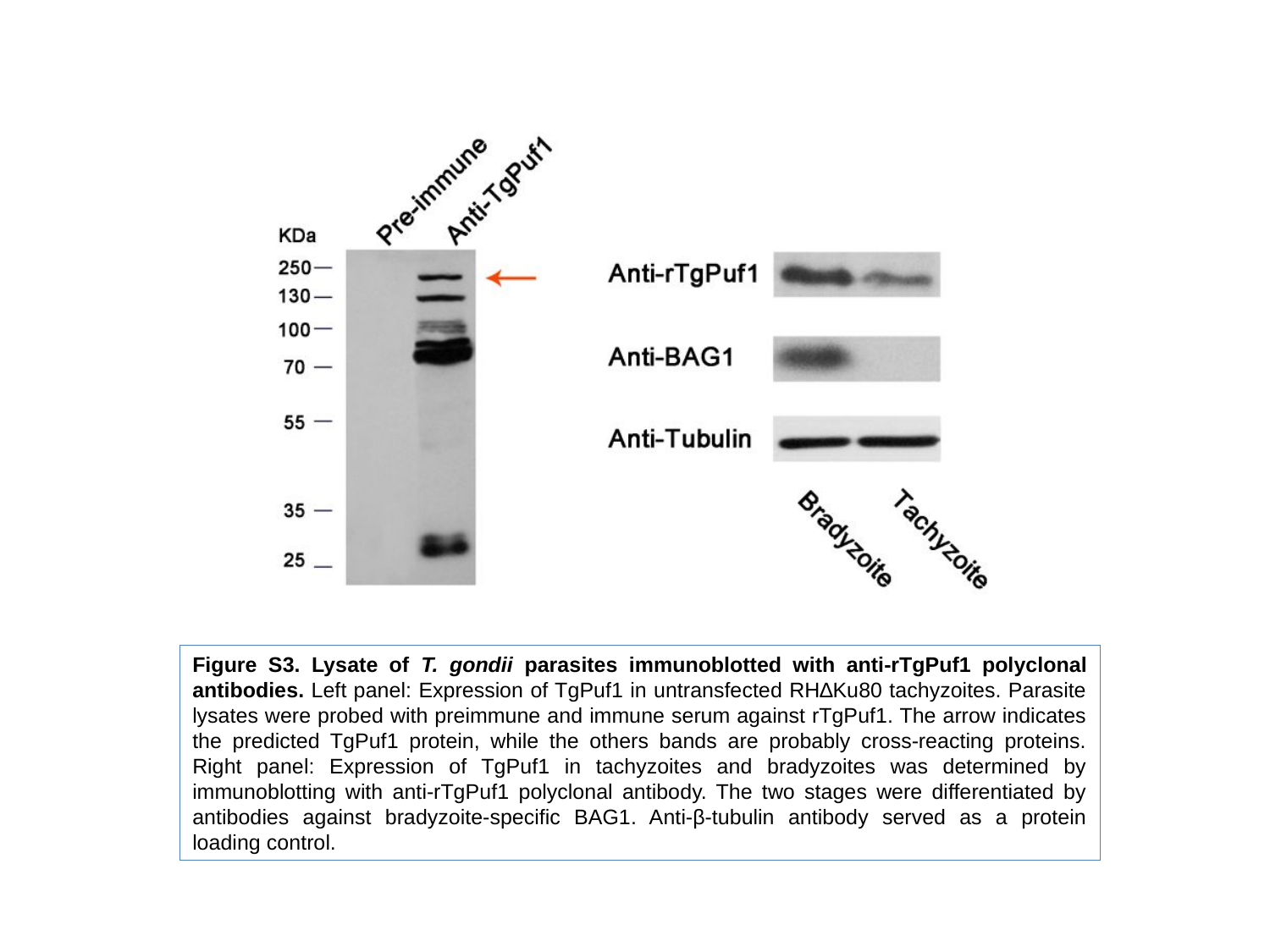

Figure S3. Lysate of T. gondii parasites immunoblotted with anti-rTgPuf1 polyclonal antibodies. Left panel: Expression of TgPuf1 in untransfected RH∆Ku80 tachyzoites. Parasite lysates were probed with preimmune and immune serum against rTgPuf1. The arrow indicates the predicted TgPuf1 protein, while the others bands are probably cross-reacting proteins. Right panel: Expression of TgPuf1 in tachyzoites and bradyzoites was determined by immunoblotting with anti-rTgPuf1 polyclonal antibody. The two stages were differentiated by antibodies against bradyzoite-specific BAG1. Anti-β-tubulin antibody served as a protein loading control.
